# Clinical factors associated with leptomeningeal disease in patients with melanoma brain metastasis

**DOI:** 10.1093/noajnl/vdag232

**Published:** 2026-09-15

**Authors:** Merve Hasanov, Christina A Faraj, Denai R Milton, Subhiksha Srinivasan, Eric A Goethe, Ian E McCutcheon, Jeffrey S Weinberg, Isabella C Glitza Oliva, Sherise D Ferguson

**Affiliations:** Department of Internal Medicine, Ohio State University Comprehensive Cancer Center, Columbus, Ohio (M.H.); Department of Neurosurgery, University of Texas MD Anderson Cancer Center, Houston, Texas (C.A.F., S.S., E.A.G., I.E.M., J.S.W., S.D.F.); Department of Biostatistics, University of Texas MD Anderson Cancer Center, Houston, Texas (D.R.M.); Department of Melanoma Medical Oncology, University of Texas MD Anderson Cancer Center, Houston, Texas (I.C.G.O.); Department of Internal Medicine, Ohio State University Comprehensive Cancer Center, Columbus, Ohio (M.H.); Department of Neurosurgery, University of Texas MD Anderson Cancer Center, Houston, Texas (C.A.F., S.S., E.A.G., I.E.M., J.S.W., S.D.F.); Department of Biostatistics, University of Texas MD Anderson Cancer Center, Houston, Texas (D.R.M.); Department of Melanoma Medical Oncology, University of Texas MD Anderson Cancer Center, Houston, Texas (I.C.G.O.); Department of Internal Medicine, Ohio State University Comprehensive Cancer Center, Columbus, Ohio (M.H.); Department of Neurosurgery, University of Texas MD Anderson Cancer Center, Houston, Texas (C.A.F., S.S., E.A.G., I.E.M., J.S.W., S.D.F.); Department of Biostatistics, University of Texas MD Anderson Cancer Center, Houston, Texas (D.R.M.); Department of Melanoma Medical Oncology, University of Texas MD Anderson Cancer Center, Houston, Texas (I.C.G.O.); Department of Internal Medicine, Ohio State University Comprehensive Cancer Center, Columbus, Ohio (M.H.); Department of Neurosurgery, University of Texas MD Anderson Cancer Center, Houston, Texas (C.A.F., S.S., E.A.G., I.E.M., J.S.W., S.D.F.); Department of Biostatistics, University of Texas MD Anderson Cancer Center, Houston, Texas (D.R.M.); Department of Melanoma Medical Oncology, University of Texas MD Anderson Cancer Center, Houston, Texas (I.C.G.O.); Department of Internal Medicine, Ohio State University Comprehensive Cancer Center, Columbus, Ohio (M.H.); Department of Neurosurgery, University of Texas MD Anderson Cancer Center, Houston, Texas (C.A.F., S.S., E.A.G., I.E.M., J.S.W., S.D.F.); Department of Biostatistics, University of Texas MD Anderson Cancer Center, Houston, Texas (D.R.M.); Department of Melanoma Medical Oncology, University of Texas MD Anderson Cancer Center, Houston, Texas (I.C.G.O.); Department of Internal Medicine, Ohio State University Comprehensive Cancer Center, Columbus, Ohio (M.H.); Department of Neurosurgery, University of Texas MD Anderson Cancer Center, Houston, Texas (C.A.F., S.S., E.A.G., I.E.M., J.S.W., S.D.F.); Department of Biostatistics, University of Texas MD Anderson Cancer Center, Houston, Texas (D.R.M.); Department of Melanoma Medical Oncology, University of Texas MD Anderson Cancer Center, Houston, Texas (I.C.G.O.); Department of Internal Medicine, Ohio State University Comprehensive Cancer Center, Columbus, Ohio (M.H.); Department of Neurosurgery, University of Texas MD Anderson Cancer Center, Houston, Texas (C.A.F., S.S., E.A.G., I.E.M., J.S.W., S.D.F.); Department of Biostatistics, University of Texas MD Anderson Cancer Center, Houston, Texas (D.R.M.); Department of Melanoma Medical Oncology, University of Texas MD Anderson Cancer Center, Houston, Texas (I.C.G.O.); Department of Internal Medicine, Ohio State University Comprehensive Cancer Center, Columbus, Ohio (M.H.); Department of Neurosurgery, University of Texas MD Anderson Cancer Center, Houston, Texas (C.A.F., S.S., E.A.G., I.E.M., J.S.W., S.D.F.); Department of Biostatistics, University of Texas MD Anderson Cancer Center, Houston, Texas (D.R.M.); Department of Melanoma Medical Oncology, University of Texas MD Anderson Cancer Center, Houston, Texas (I.C.G.O.); Department of Internal Medicine, Ohio State University Comprehensive Cancer Center, Columbus, Ohio (M.H.); Department of Neurosurgery, University of Texas MD Anderson Cancer Center, Houston, Texas (C.A.F., S.S., E.A.G., I.E.M., J.S.W., S.D.F.); Department of Biostatistics, University of Texas MD Anderson Cancer Center, Houston, Texas (D.R.M.); Department of Melanoma Medical Oncology, University of Texas MD Anderson Cancer Center, Houston, Texas (I.C.G.O.)

**Keywords:** brain metastasis, craniotomy, leptomeningeal disease, melanoma

## Abstract

**Background:**

Leptomeningeal disease (LMD) is a severe manifestation of metastatic cancer with limited treatment options and a poor prognosis. Melanoma has one of the highest rates of leptomeningeal spread among solid tumors. Given the associated morbidity and mortality, identifying clinical factors linked to LMD in patients with melanoma brain metastases (MBM) is crucial.

**Methods:**

We conducted a retrospective study of 829 MBM patients diagnosed at MD Anderson Cancer Center (2009-2018). The primary outcome measure was time from MBM to LMD. Cumulative incidence of LMD was determined using the competing risks method, where death was the competing risk. Differences in cumulative incidences were assessed using Gray’s test, and associations between measures of interest and cumulative incidence were determined using proportional sub-distribution hazards models.

**Results:**

Among 829 patients, 129 (16%) developed LMD (median follow-up: 12.1 months, range: 0.0-156.2 months). Median time from MBM diagnosis to LMD was 5.5 months. Of those with known mutation status (*n* = 713), BRAF V600 mutation was present in 74% of patients who developed LMD versus 50% in those who did not (*P* < .001). Multivariable analysis (MVA) showed BRAF V600 mutation (*P* = .009) and craniotomy (*P* = .035) were associated with the development of LMD. In a sub-analysis of BRAF V600 mutant patients (*n* = 380), MVA revealed that primary melanoma site (upper extremity vs head/neck) and number of MBMs at diagnosis were associated with LMD development.

**Conclusion:**

LMD is a dire consequence of advanced melanoma, yet the clinical features associated with its development are not fully characterized. Identifying clinical predictors may guide management of high-risk populations.

Key Points

- LMD developed in 16% of patients, with a median onset of 5.5 months after MBM diagnosis.
- BRAF V600 mutation and craniotomy as initial MBM treatment are associated with increased LMD risk in patients with MBMs.
- Primary tumor site and number of MBMs predict LMD risk in BRAF V600-mutant cases.

Importance of the Study

Melanoma brain metastasis (MBM) affects 40%-60% of patients with advanced melanoma and carries a poor prognosis. Leptomeningeal disease (LMD), a devastating complication involving tumor spread to the leptomeninges, further worsens outcomes and is increasingly recognized in MBM patients, possibly due to prolonged survival from improved local and systemic therapies. Despite this, limited data exist on predictors of LMD in this population. In this large single-institution retrospective study of 829 patients with MBM, we found a 16% incidence of LMD. BRAF V600 mutations and craniotomy as the initial treatment were independently associated with increased LMD risk on multivariable analysis. In an exploratory subgroup analysis of BRAF V600-mutant patients, having 4 to 10 brain metastases was associated with increased LMD risk. These associations are hypothesis-generating and do not establish causal mechanisms. By characterizing clinical and molecular factors associated with LMD risk, this study contributes to an emerging evidence base that may help guide the design of future prospective studies.

Melanoma brain metastasis (MBM) occurs in around 40%-60% of patients with advanced melanoma and has a dismal prognosis.^1^^,^^2^ Although our recent study showed improvement in outcomes of MBM patients over time with advancement of systemic therapies and local therapies,^3^ the presence of leptomeningeal disease (LMD) remains the worst prognostic factor in the current treatment era.^4^ LMD is the spread of malignancy to the leptomeninges, protective layers that surround the brain and spinal cord.^1^ Melanoma is one of the primary tumors most commonly associated with LMD.^2^^,^^4^ Recent literature suggests that the incidence of LMD is rising, potentially due to advancements in local and systemic therapies that have extended overall survival and improved control of both BMs and systemic disease.^2^^,^^4^^,^^5^ Despite these therapeutic advances, LMD in melanoma continues to portend a grim outlook, with a median overall survival of only 3.5 months even with treatment.^6^^,^^7^

Although only about 5% of patients with melanoma ultimately receive a diagnosis of LMD, coexisting MBM has been observed in around 70% of these patients.^1^ Notably, a substantial proportion of LMD cases have been observed in BRAF V600-mutant melanoma patients,^6^^,^^8^ and some case studies have even investigated the utility of targeted BRAF inhibitors in LMD from melanoma.^9^ This investigation is particularly meaningful in melanoma, a population in which a significant proportion (around 50%) harbor BRAF V600 mutations.^9^^,^^10^

Several factors have been associated with improved outcomes in patients with LMD, including absence of active systemic disease, receipt of LMD-directed treatment, and use of targeted or intrathecal therapies.^6^ However, less robust data exist on predictors of LMD development specifically in patients with established MBMs, and findings remain inconsistent across studies. Surgical resection has emerged as a potential risk factor, with previous reports identifying associations between LMD and various features such as piecemeal resections, posterior fossa location, multiplicity of metastases, and the presence of hemorrhagic or cystic characteristics.^7^^,^^11-16^

In this large retrospective study from a single institution, we examine factors contributing to the development of LMD in patients with MBM.

## Methods

### Patients and Data

This is a single-center retrospective cohort study that was conducted under an institutional review board-approved protocol to examine the factors associated with LMD in patients diagnosed with MBM. All consecutive patients diagnosed with MBM from 2009 to 2018 inclusive, and who received their initial MBM treatment at the University of Texas MD Anderson Cancer Center, were considered for the study. Exclusion criteria included uveal and mucosal melanoma, concurrent non-melanoma cancers, initial MBM treatment at another center, or unavailable CNS imaging from which the initial diagnosis of MBM was made. The data are anonymous, and the requirement for informed consent was therefore waived.

Extensive patient, disease, and treatment data were collected. These included gender, age at MBM diagnosis, primary melanoma features (primary tumor site, histologic subtype, Breslow thickness, ulceration, mitotic rate, lymphovascular and perineural invasion), BRAF V600 and NRAS mutation status, features at initial MBM diagnosis (presence of symptoms, number and maximum size of MBMs, LDH, extracranial disease status, use of steroids at initial MBM diagnosis), treatments prior to MBM diagnosis (prior immunotherapy and targeted therapy), and initial MBM treatment (SRS, WBRT, chemotherapy, immunotherapy, targeted therapy, craniotomy). Details regarding craniotomy, including number, location (posterior fossa, intraventricular, dural-based), characteristics (hemorrhagic or cystic) of the tumors resected, and resection technique (en bloc versus piecemeal) were also collected. Initial MBM treatment was defined as any treatment given for MBM management within 6 weeks of initial MBM diagnosis. The immunotherapy category included single-agent ipilimumab, nivolumab, pembrolizumab, or interleukin-2 (IL-2), and ipilimumab and nivolumab in combination. Targeted therapies included single-agent BRAF (BRAFi), single-agent MEK (MEKi), combination BRAFi + MEKi, and c-KIT (KITi) inhibitors. LMD diagnosis was determined from review of cerebrospinal fluid (CSF) pathological analysis and/or radiology reports of brain and/or spine MRIs detailing the presence of LMD. Either the presence of malignant cells in CSF or radiographic evidence of LMD was considered sufficient for LMD diagnosis.

### Clinical Outcome Assessment and Statistical Analysis

The primary endpoint for the study was time-to-LMD, computed from the date of MBM diagnosis to the date of LMD diagnosis. The cumulative incidence of LMD was determined using the competing risks method. The competing risk included was death, and patients who did not develop LMD and were still alive at the last follow-up date were censored. Differences in cumulative incidences between groups were assessed using Gray’s test,^17^ while associations between measures of interest and the cumulative incidence were determined using proportional subdistribution hazards regression models.^18^ Variables whose *P*-value was ≤ .05 on univariable analyses were included in the multivariable model. All statistical analyses were performed using SAS 9.4 for Windows (Copyright © 2002-2012 by SAS Institute Inc.). All statistical tests used a significance level of 5%. No adjustments for multiple testing were made.

## Results

### Demographics, Disease, Treatment Characteristics and Time-to-LMD

Patients (*n* = 829) who were diagnosed with and received initial treatment for MBM at the University of Texas MD Anderson Cancer Center were included in the study. Over a median follow-up of 12.1 (0.0-156.2) months, 129 (15.6%) patients developed LMD. The median time from initial melanoma to LMD diagnosis was 42.4 (range, 2.1-283.5) months, whereas time from MBM to LMD diagnosis was 5.5 (0-141) months. The median overall survival post-LMD was 3.2 (range, 2.4-4.4) months.

Patient demographics, primary melanoma, and MBM disease characteristics, treatment prior to MBM, initial MBM treatment types, and details are provided in Table 1 for both cohorts combined and for the subsets of patients with LMD and patients without LMD. Patients with LMD were significantly younger at the time of MBM diagnosis (median age 55.2 vs 61.7 years, *P* = .002). A BRAF V600 mutation was more common in LMD patients (74% vs 50%, *P* < .001), while NRAS mutations were less common (11% vs 23%, *P* = .010).

**Table 1.** Patient, disease, and treatment characteristics of MBM patients

Measure	All patients (*N* = 829)	Patients w/LMD (*N* = 129)	Patients w/o LMD (*N* = 700)	*P*-value**^a^**
Follow-up for all patients (months), median (range)	12.1 (0.0-156.2)	10.3 (0.9-133.4)	12.3 (0.0-156.2)	**.011^b^**
Gender, *n* (%)				
Female	552 (67)	82 (64)	470 (67)	.43
Male	277 (33)	47 (36)	230 (33)	
Age at MBM diagnosis (years), median (range)	61.1 (15.6-93.6)	55.2 (23.9-86.7)	61.7 (15.6-93.6)	**.002^b^**
BRAF V600 mutation, *n* (%)				
Yes	380 (53)	81 (74)	299 (50)	**<.001**
No	333 (47)	28 (26)	305 (50)	
Unknown	116	20	96	
NRAS status, *n* (%)				
Mutation	130 (21)	10 (11)	120 (23)	**.010**
Wild type	490 (79)	82 (89)	408 (77)	
Unknown	209	37	172	
LDH at MBM diagnosis, *n* (%)				
Not elevated	549 (66)	90 (70)	459 (66)	.35
Elevated	280 (34)	39 (30)	241 (34)	
Primary tumor site, *n* (%)				
Scalp	92 (15)	11 (11)	81 (15)	.38
Head and neck (non-scalp)	109 (17)	14 (14)	95 (18)	
Upper extremity	111 (18)	17 (17)	94 (18)	
Trunk	247 (39)	45 (44)	202 (38)	
Lower extremity	71 (11)	15 (15)	56 (11)	
Unknown	199	27	172	
Melanoma subtype, *n* (%)				
Superficial spreading	227 (31)	47 (42)	180 (29)	**.008**
Cutaneous	169 (23)	29 (26)	140 (23)	.47
Nodular	136 (19)	15 (13)	121 (20)	.12
Lentigo maligna	39 (5)	3 (3)	36 (6)	.25^c^
Acral lentiginous	50 (7)	7 (6)	43 (7)	1.00^c^
Unknown primary	112 (15)	12 (11)	100 (16)	.13
Breslow thickness (mm), median (range)	2.5 (0.0-29.0)	2.3 (0.4-25.2)	2.5 (0.0-29.0)	.28^b^
Ulceration, *n* (%)	206 (40)	35 (41)	171 (40)	.96
Mitotic rate (/mm^2^), median (range)	6.0 (0.0-58.0)	5.0 (1.0-34.0)	6.0 (0.0-58.0)	**.022^b^**
Mitotic rate, *n* (%)				
0-1/mm^2^	85 (17)	15 (18)	70 (17)	.06
2-6/mm^2^	177 (36)	39 (46)	138 (33)	
>6/mm^2^	235 (47)	31 (36)	204 (50)	
Unknown	332	44	288	
Lymphovascular invasion, *n* (%)	153 (32)	23 (27)	130 (33)	.27
Perineural invasion, *n* (%)	62 (13)	10 (12)	52 (13)	.81
Number of brain metastases at diagnosis, median (range)	2 (1-200)	2 (1-150)	2 (1-200)	.22^b^
Number of brain metastases at diagnosis, *n* (%)				
1	353 (43)	48 (38)	305 (44)	.51
2-3	231 (28)	40 (31)	191 (27)	
4-10	164 (20)	25 (20)	139 (20)	
>10	79 (10)	15 (12)	64 (9)	
Unknown	2	1	1	
Brain metastasis maximum size (cm), median (range)	1.0 (0.1-11.4)	1.2 (0.2-6.0)	1.0 (0.1-11.4)	.14^b^
Brain metastasis maximum size, *n* (%)				
≤3.0 cm	714 (90)	101 (86)	613 (91)	.15
>3.0 cm	79 (10)	16 (14)	63 (9)	
Unknown	36	12	24	
Symptomatic brain metastasis detection, *n* (%)	219 (26)	39 (30)	180 (26)	.28
Prior immunotherapy, *n* (%)	267 (32)	45 (35)	222 (32)	.48
Prior targeted therapy, *n* (%)	163 (20)	39 (30)	124 (18)	**.001**
Steroid use (yes/periprocedural only), *n* (%)	332 (41)	56 (45)	276 (40)	.33
Extracranial stage IV disease status at MBM diagnosis, *n* (%)				
Uncontrolled	160 (19)	32 (25)	128 (18)	.11
Controlled	597 (73)	84 (65)	513 (74)	
No	65 (8)	13 (10)	52 (8)	
Unknown	7	0	7	
Initial MBM treatment				
SRS, *n* (%)	459 (55)	59 (46)	400 (57)	**.017**
WBRT, *n* (%)	140 (17)	24 (19)	116 (17)	.57
Systemic, *n* (%)	467 (56)	72 (56)	395 (56)	.90
Chemotherapy, *n* (%)	149 (18)	20 (16)	129 (18)	.43
Immunotherapy, *n* (%)	229 (28)	27 (21)	202 (29)	.06
Targeted therapy, *n* (%)	151 (18)	38 (29)	113 (16)	**<.001**
Craniotomy, *n* (%)	122 (15)	28 (22)	94 (13)	**.015**
Number of tumors resected, median (range)	1 (1-4)	1 (1-2)	1 (1-4)	.24^b^
Number of tumors resected, *n* (%)				
1	104 (87)	27 (93)	77 (85)	.24
>1	16 (13)	2 (7)	14 (15)	
Posterior fossa lesion resected, *n* (%)	11 (9)	6 (21)	5 (5)	**.014**
Intraventricular lesion resected, *n* (%)	1 (1)	0	1 (1)	1.00^c^
Dural-based lesion resected, *n* (%)	4 (3)	1 (3)	3 (3)	1.00^c^
Cystic lesion resected, *n* (%)	14 (12)	3 (10)	11 (12)	.80
Hemorrhagic lesion resected, *n* (%)	87 (73)	17 (59)	70 (77)	.055
Resection method, *n* (%)				
En bloc	59 (50)	15 (54)	44 (49)	.70
Piecemeal	58 (50)	13 (46)	45 (51)	
Unknown	3	1	2	

a Chi-squared test.

b Wilcoxon rank-sum test.

c Fisher’s exact test.

Abbreviations: LDH, lactate dehydrogenase; MBM, melanoma brain metastasis; SRS, stereotactic radiosurgery; WBRT, whole brain radiation therapy. The bold values represent statistical significance.

Certain melanoma subtypes, particularly superficial spreading melanoma, were more frequent in the LMD group (42% vs 29%, *P* = .008), while other subtypes showed no significant difference. Although the median mitotic rate of the primary melanoma was slightly lower in the LMD group (5.0 vs 6.0, *P* = .022), there were no significant differences in ulceration, lymphovascular invasion, perineural invasion, or the number and size of brain metastases at diagnosis. The use of prior targeted therapy was significantly more frequent in LMD patients (30% vs 18%, *P* = .001), as was the use of targeted therapy as initial MBM treatment (29% vs 16%, *P* < .001). LMD patients were also more likely to have undergone craniotomy (22% vs 13%, *P* = .015) and less likely to have received initial treatment with stereotactic radiosurgery (SRS) (46% vs 57%, *P* = .017).

Among those who underwent surgery, resected posterior fossa lesions were more frequent in the LMD group (21% vs 5%, *P* = .014). Other surgical features, such as the number of tumors resected, presence of cystic or hemorrhagic lesions, and resection technique (en bloc vs piecemeal), did not differ significantly in incidence.

### Univariate and Multivariable Analysis of Predictors of LMD in MBM Patients

The univariate cumulative incidence of LMD for 829 MBM patients was 11% at 1 year, 14% at 2 years, 15% at 5 years, 15% at 10 years, and 22% at the end of the assessment period (Figure 1A).

**Figure 1.**
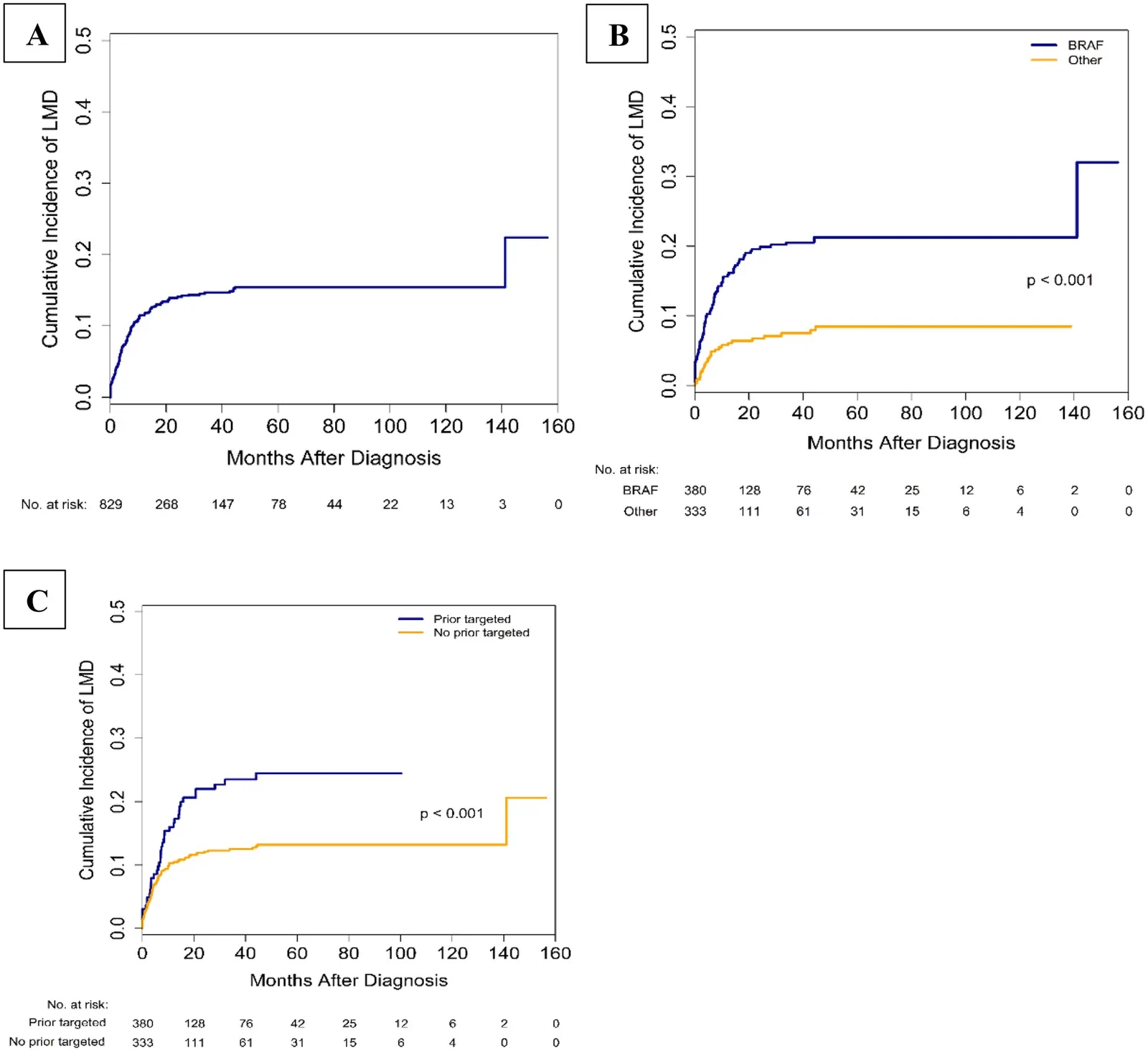
Univariate cumulative incidence of LMD (A) for the overall cohort, and stratified by (B) BRAF V600 mutation status (*P* < .001) and (C) prior targeted therapy (*P* < .001).

In the univariate analysis (Table 2), several factors were significantly associated with LMD risk. Patients harboring a BRAF V600 mutation (HR 2.83, 95% CI: 1.82-4.40, *P* < .001) and those who received targeted therapy prior to MBM diagnosis (HR 1.89, 95% CI: 1.30-2.76, *P* < .001) had significantly higher LMD risk. Stratified cumulative incidence curves by BRAF V600 mutation status and receipt of targeted therapy prior to MBM diagnosis are shown in Figure 1B and C, respectively. Among initial treatments for MBM, both targeted therapy (HR 2.09, 95% CI: 1.41-3.12, *P* < .001) and craniotomy (HR 1.85, 95% CI: 1.17-2.92, *P* = .009) were associated with increased LMD risk. Conversely, older age (HR 0.98, 95% CI: 0.97-0.99, *P* = .003), presence of an NRAS mutation (HR 0.46, 95% CI: 0.24-0.88, *P* = .019), higher mitotic rate (HR 0.96, 95% CI: 0.93-0.99, *P* = .010), and nodular melanoma subtype compared to superficial spreading (HR 0.53, 95% CI: 0.29-0.96, *P* = .036) were all associated with a lower risk of LMD.

**Table 2.** Univariate and multivariable analysis of cumulative incidence of LMD in MBM patients

	Univariate		Multivariable	
Measure	Hazard ratio (95% CI)	*P*-value	Hazard ratio (95% CI)	*P*-value
Gender				
Female vs male	1.03 (0.71, 1.48)	.90	NI	
Age at MBM diagnosis *(continuous)*	0.98 (0.97, 0.99)	**.003**	0.99 (0.98, 1.00)	.058
BRAF V600 mutation				
Mutated vs wild-type	2.83 (1.82, 4.40)	**<.001**	2.29 (1.23, 4.26)	**.009**
Unknown vs wild-type			1.66 (0.74, 3.69)	.22
NRAS status				
Mutated vs wild-type	0.46 (0.24, 0.88)	**.019**	0.99 (0.46, 2.11)	.97
Unknown vs wild-type			1.19 (0.68, 2.06)	.54
LDH at MBM diagnosis				
Elevated vs not elevated	0.89 (0.61, 1.30)	.55	NI	
Primary tumor site				
Scalp vs upper extremity	0.46 (0.20, 1.06)	.07	NI	
Head and neck (non-scalp) vs upper extremity	0.58 (0.28, 1.20)	.14		
Trunk vs upper extremity	0.83 (0.47, 1.47)	.53		
Lower extremity vs upper extremity	0.76 (0.38, 1.50)	.43		
Melanoma subtype				
Cutaneous vs superficial spreading	0.84 (0.53, 1.35)	.47	NI	
Nodular vs superficial spreading	0.53 (0.29, 0.96)	**.036**		
Lentigo maligna vs superficial spreading	0.37 (0.12, 1.20)	.10		
Acral lentiginous vs superficial spreading	0.73 (0.33, 1.61)	.44		
Unknown primary vs superficial spreading	0.56 (0.29, 1.07)	.08		
Breslow thickness (mm)	0.97 (0.89, 1.05)	.46	NI	
Ulceration				
Present vs absent	1.00 (0.64, 1.56)	.99	NI	
Mitotic rate *(continuous)*	0.96 (0.93, 0.99)	**.010**	NI	
Mitotic rate				
2-6/mm^2^ vs 0-1/mm^2^	1.25 (0.70, 2.26)	.45	1.13 (0.59, 2.15)	.71
>6/mm^2^ vs 0-1/mm^2^	0.70 (0.38, 1.30)	.26	0.71 (0.37, 1.36)	.30
Unknown vs 0-1/mm^2^			0.69 (0.37, 1.30)	.25
Lymphovascular invasion				
Present vs absent	0.74 (0.45, 1.22)	.24	NI	
Perineural invasion				
Present vs absent	0.92 (0.46, 1.85)	.82	NI	
Number of brain metastases at diagnosis *(continuous)*	1.01 (1.00, 1.02)	.17	NI	
Number of brain metastases at diagnosis				
2-3 vs 1	1.27 (0.84, 1.94)	.26	NI	
4-10 vs 1	1.06 (0.64, 1.75)	.82		
>10 vs 1	1.15 (0.61, 2.17)	.67		
Brain metastasis maximum size *(continuous)*	1.12 (0.99, 1.26)	.08	1.09 (0.91, 1.29)	.35
Brain metastasis maximum size				
>3.0 cm vs ≤3.0 cm	1.50 (0.87, 2.57)	.14	NI	
Brain metastasis detection type				
Symptomatic vs asymptomatic	1.33 (0.91, 1.94)	.14	NI	
Prior immunotherapy				
Yes vs no	1.14 (0.79, 1.64)	.49	NI	
Prior targeted therapy				
Yes vs no	1.89 (1.30, 2.76)	**<.001**	1.39 (0.87, 2.22)	.17
Steroid use				
Yes vs no	1.24 (0.85, 1.81)	.27	NI	
Extracranial stage IV disease status at MBM diagnosis				
Controlled vs no	1.01 (0.54, 1.90)	.98	NI	
Uncontrolled vs no	0.67 (0.37, 1.20)	.18		
Initial MBM treatment (yes vs no)^a^				
SRS	0.74 (0.51, 1.07)	.11	NI	
WBRT	0.65 (0.36, 1.18)	.15	NI	
Systemic	0.99 (0.69, 1.43)	.96	NI	
Chemotherapy	0.78 (0.45, 1.32)	.35	NI	
Immunotherapy	0.64 (0.40, 1.03)	.07	0.74 (0.44, 1.24)	.25
Targeted therapy	2.09 (1.41, 3.12)	**<.001**	1.45 (0.90, 2.34)	.12
Craniotomy	1.85 (1.17, 2.92)	**.009**	1.90 (1.04, 3.45)	**.035**
Number of tumors resected				
>1 vs 1	0.28 (0.04, 2.02)	.21	NI	
Posterior fossa lesion resected				
Yes vs no	3.15 (0.94, 10.51)	.06	NI	
Cystic lesion resected				
Yes vs no	1.02 (0.32, 3.26)	.97	NI	
Hemorrhagic lesion resected				
Yes vs no	0.60 (0.27, 1.33)	.21	NI	
Resection method				
En bloc vs piecemeal	1.00 (0.47, 2.16)	.99	NI	

a Included in the model as a time-dependent covariate.

Abbreviations: LDH, lactate dehydrogenase; MBM, melanoma brain metastasis; NI, not included in the multivariable model; SRS, stereotactic radiosurgery; WBRT, whole brain radiation therapy. The bold values represent statistical significance.

In the multivariable model adjusting for age, BRAF V600 and NRAS mutation statuses, mitotic rate, maximum brain metastasis size, prior targeted therapy, and initial MBM treatments (including craniotomy, immunotherapy, and targeted therapy) (Table 2), two variables remained significantly associated with LMD risk: BRAF V600 mutation (HR 2.29, 95% CI: 1.23-4.26, *P* = .009) and craniotomy as initial MBM treatment (HR 1.90, 95% CI: 1.04-3.45, *P* = .035).

### Subgroup Analysis in BRAF V600 Mutated MBM Patients

We further conducted subgroup univariate and multivariable analyses among patients with BRAF V600-mutated MBM to identify risk factors for LMD, given the notably higher incidence of LMD in this population.

In the univariate analysis (Table 3), patients with primary melanomas located on the scalp (HR 0.37, 95% CI: 0.14-0.95, *P* = .040) or head and neck (HR 0.29, 95% CI: 0.10-0.88, *P* = .029) had a significantly lower risk of developing LMD compared with those with primary tumors on the upper extremity. Factors associated with a higher risk of LMD included having 2 to 3 MBMs at diagnosis (HR 1.79, 95% CI: 1.04-3.10, *P* = .036) versus a single lesion, larger maximum brain metastasis size (HR 1.30, 95% CI: 1.05-1.61, *P* = .016), and the presence of symptomatic brain metastases (HR 1.71, 95% CI: 1.07-2.73, *P* = .025).

**Table 3.** Subgroup univariate and multivariable analysis of cumulative incidence of LMD in MBM patients with BRAF V600 mutation

	Univariate		Multivariable	
Measure	Hazard ratio (95% CI)	*P*-value	Hazard ratio (95% CI)	*P*-value
Gender				
Female vs male	0.85 (0.54, 1.35)	.50	NI	
Age at MBM diagnosis *(continuous)*	0.99 (0.98, 1.01)	.31	NI	
LDH at MBM diagnosis				
Elevated vs not elevated	0.99 (0.62, 1.57)	.96	NI	
Primary tumor site				
Scalp vs upper extremity	0.37 (0.14, 0.95)	**.040**	0.39 (0.13, 1.14)	.09
Head and neck (non-scalp) vs upper extremity	0.29 (0.10, 0.88)	**.029**	0.29 (0.09, 0.93)	**.038**
Trunk vs upper extremity	0.59 (0.29, 1.21)	.15	0.57 (0.25, 1.27)	.17
Lower extremity vs upper extremity	0.50 (0.20, 1.24)	.14	0.49 (0.16, 1.45)	.20
Melanoma subtype				
Cutaneous vs superficial spreading	0.86 (0.49, 1.49)	.58	NI	
Nodular vs superficial spreading	0.50 (0.24, 1.06)	.07		
Lentigo maligna vs superficial spreading	0.54 (0.07, 4.29)	.56		
Acral lentiginous vs superficial spreading	1.54 (0.50, 4.70)	.45		
Unknown primary vs superficial spreading	0.47 (0.19, 1.15)	.10		
Breslow thickness (mm)	0.98 (0.90, 1.07)	.71	NI	
Ulceration				
Present vs absent	1.17 (0.68, 2.01)	.56	NI	
Mitotic rate *(continuous)*	0.96 (0.93, 1.00)	**.030**	NI	
Mitotic rate				
2-6/mm^2^ vs 0-1/mm^2^	1.21 (0.58, 2.52)	.62	NI	
>6/mm^2^ vs 0-1/mm^2^	0.78 (0.38, 1.63)	.51		
Lymphovascular invasion				
Present vs absent	0.73 (0.41, 1.30)	.29	NI	
Perineural invasion				
Present vs absent	1.31 (0.52, 3.30)	.57	NI	
Number of brain metastases at diagnosis *(continuous)*	1.01 (1.00, 1.01)	.25	NI	
Number of brain metastases at diagnosis				
2-3 vs 1	1.79 (1.04, 3.10)	**.036**	1.89 (0.95, 3.78)	.07
4-10 vs 1	1.75 (0.95, 3.20)	.07	2.20 (1.05, 4.58)	**.036**
>10 vs 1	1.42 (0.66, 3.09)	.37	1.11 (0.40, 3.11)	.84
Brain metastasis maximum size *(continuous)*	1.30 (1.05, 1.61)	**.016**	1.11 (0.82, 1.51)	.48
Brain metastasis maximum size				
>3.0 cm vs ≤3.0 cm	1.87 (0.86, 4.08)	.11	NI	
Brain metastasis detection type				
Symptomatic vs asymptomatic	1.71 (1.07, 2.73)	**.025**	1.51 (0.82, 2.78)	.19
Prior immunotherapy				
Yes vs no	1.49 (0.95, 2.33)	.08	1.52 (0.85, 2.71)	.16
Prior targeted therapy				
Yes vs no	1.21 (0.77, 1.88)	.41	NI	
Steroid use				
Yes vs no	1.43 (0.88, 2.31)	.15	NI	
Extracranial stage IV disease status at MBM diagnosis				
Controlled vs no	0.88 (0.41, 1.87)	.73	NI	
Uncontrolled vs no	0.76 (0.39, 1.48)	.42		
Initial MBM treatment (yes vs no)^a^				
SRS	1.01 (0.64, 1.60)	.96	NI	
WBRT	0.60 (0.27, 1.31)	.20	NI	
Systemic	0.75 (0.48, 1.19)	.22	NI	
Chemotherapy	0.71 (0.34, 1.49)	.36	NI	
Immunotherapy	0.58 (0.31, 1.08)	.09	0.54 (0.23, 1.25)	.15
Targeted therapy	1.48 (0.94, 2.34)	.09	1.11 (0.60, 2.05)	.73
Craniotomy	1.98 (0.95, 4.15)	.07	1.29 (0.50, 3.32)	.59
Cystic lesion resected				
Yes vs no	1.60 (0.32, 8.03)	.57	NI	
Hemorrhagic lesion resected				
Yes vs no	1.51 (0.44, 5.25)	.51	NI	
Resection method				
En bloc vs piecemeal	0.52 (0.15, 1.78)	.30	NI	

a Included in the model as a time-dependent covariate.

Abbreviations: LDH, lactate dehydrogenase; MBM, melanoma brain metastasis; NI, not included in the multivariable model; SRS, stereotactic radiosurgery; WBRT, whole brain radiation therapy. The bold values represent statistical significance.

In the multivariable model (Table 3), head and neck (non-scalp) primary tumors remained significantly associated with reduced LMD risk compared to upper extremity primary site (HR 0.29, 95% CI: 0.09-0.93, *P* = .038). Conversely, patients with 4 to 10 MBMs had a significantly increased risk of LMD (vs 1 lesion; HR 2.20, 95% CI: 1.05-4.58, *P* = .036). Variables such as metastasis size and symptomatic presentation that were significant in univariate analysis did not remain so after multivariable adjustment. Furthermore, prior immunotherapy and initial MBM treatments (craniotomy, immunotherapy, targeted therapy) were not significantly associated with LMD risk.

## Discussion

LMD occurs frequently in patients with melanoma brain metastases. In our institutional series of 829 patients initially treated for MBM, we found an overall LMD rate of 16%, which is consistent with prior reports ranging from 5% to 31%.^7^^,^^11^^,^^13-15^^,^^19^^,^^20^ The apparent plateau in cumulative LMD incidence beyond the early follow-up period likely reflects the substantial competing risk of mortality in patients with melanoma brain metastases, which limits the population remaining at risk for late LMD, as well as potential censoring from limited long-term follow-up.

In multivariable analysis, two factors were independently associated with LMD in our cohort: the presence of a BRAF V600 mutation and craniotomy as the initial modality of MBM treatment. Within the BRAF V600-mutant subgroup, having 4 to 10 MBMs correlated with higher LMD risk. The median time from MBM to LMD diagnosis in our cohort was 5.5 months. Prior studies have revealed a particularly short time interval between the diagnosis of advanced melanoma or MBM and the subsequent development of LMD.^8^^,^^21^ One retrospective analysis reported a median time of 8.5 months from unresectable metastatic melanoma to LMD development, while another report observed a more rapid progression (median, 2.4 months) from MBM to LMD diagnosis.^8^^,^^21^ Due to the overall poor prognosis of LMD arising from melanoma, it is prudent to examine factors contributing to accelerated versus gradual LMD development.

The association between craniotomy and LMD in our study is likely multifactorial rather than solely dependent on tumor size. Although one might surmise that craniotomy is generally done disproportionately for larger tumors, the absence of an independent association between tumor size and LMD after multivariable adjustment suggests that tumor size alone is unlikely to fully account for the observed craniotomy-LMD relationship. Residual confounding related to disease severity or surgical selection cannot be excluded. Within surgical subgroups, tumor volume may still independently predict LMD, as suggested by Morshed et al.^19^ Historical observations have indeed linked surgical resection, particularly in the posterior fossa, to LMD development.^7^^,^^22-25^ The proposed mechanism of LMD development following surgery appears distinct from hematogenous dissemination and has been hypothesized to involve direct intraoperative spillage.^19^ In our data, posterior fossa resection was more common among patients who developed LMD on descriptive comparison (21% vs 5%, *P* = .014) and was associated with an approximately 3-fold higher hazard of LMD on univariate analysis, although this did not reach statistical significance. The lack of statistical significance may reflect limited statistical power given the relatively small number of posterior fossa resections. This variable was not included in the multivariate model. Accordingly, the observed association should be interpreted cautiously as exploratory, and its independent relationship with LMD risk remains uncertain.

On the other hand, other studies did not find that surgical resection itself increased the risk of LMD.^26^^,^^27^ Instead, surgical proximity to CSF pathways, such as in dural-based and intraventricular metastasis location, metastases abutting the ventricles, and ventricular entry during surgery may contribute to LMD risk.^19^^,^^26^ In this context, the association observed in the present study between craniotomy and LMD may reflect its role as a surrogate for CSF-adjacent disease, mass effect, or other surgical-selection factors not fully captured in our retrospective modeling.

Lowe et al found that systemic chemotherapy administered at the time of initial MBM diagnosis was associated with an increased risk of LMD in non-surgical patients.^26^ As a result, chemotherapy is typically reserved for later stages of the disease, after other treatment modalities have been exhausted. Furthermore, in our study, targeted therapy administered prior to MBM diagnosis was associated with increased LMD risk in univariate analysis. Although this did not remain significant in multivariate analysis, the result may reflect a form of survival bias, whereby patients with longer follow-up durations inherently have greater opportunity to develop LMD. Alternatively, targeted therapy may allow for the emergence of more aggressive tumor clones with a propensity for LMD. In a similar fashion, Morshed et al also reported that the absence of extracranial disease at the index surgery was a main risk factor for postoperative LMD among 217 patients who underwent resection of 225 BMs followed by postoperative radiation therapy.^19^ Although our study did not find an association between extracranial disease and LMD development, this discrepancy may stem from the timing of our assessment, which focused on extracranial disease status at MBM diagnosis rather than at the time of surgery.

Our data suggest that patients with BRAF V600 mutations are more likely to develop LMD over time. Approximately 40%-60% of cutaneous melanomas harbor BRAF mutations that lead to constitutive activation of the MAPK pathway. Of these, around 90% involve a V600E substitution, although other mutations (eg, BRAF V600K and V600R) are also recognized.^28^ In the absence of BRAF inhibitors, patients with BRAF V600E-mutant melanoma tend to have worse prognoses compared to those with wild-type BRAF.^29–32^ The interpretation of the association between BRAF mutation status and LMD warrants caution. The relationship may reflect intrinsic biologic features, particularly the increased melanoma cell invasiveness by downregulating the cGMP-specific phosphodiesterase PDE5A^30^ and the heightened resistance to chemotherapy,^32^ of *BRAF V600*-mutant melanoma that predispose to LMD; however, alternative explanations are equally plausible. Patients with *BRAF V600*-mutant disease are more likely to receive effective targeted therapies and may experience prolonged survival, thereby increasing the time at risk for LMD development (survival bias). In addition, more intensive imaging surveillance and multimodal treatment exposure may contribute to earlier or more frequent detection of clinically occult LMD (treatment and detection bias). Accordingly, the observed association should be interpreted as observational and hypothesis-generating rather than indicative of a direct causal biological effect. Further prospective and multi-institutional studies incorporating treatment-era-specific and time-dependent analyses will be necessary to clarify the relative contributions of tumor biology and therapy-related factors.

While other studies have not specifically examined this subgroup, multiple brain metastases are generally recognized as a risk factor for LMD.^12-14^^,^^27^^,^^33-35^ In our cohort of patients with BRAF V600 mutations, having 4-10 MBMs independently predicted LMD; however, given that this finding arose from a subgroup analysis within a molecularly defined subset without adjustment for multiple testing, it should be regarded as exploratory and interpreted with caution. Having more than 10 lesions did not confer a greater risk of LMD, but that could be attributed to the lower sample size in this subgroup.

Metastasis size or symptomatic MBM presentation did not independently predict LMD after multivariable adjustment. This could potentially be due to confounding by overall intracranial disease burden. Disease extent—reflected most strongly by number of metastases—may be a more fundamental driver of LMD risk than individual lesion characteristics within BRAF-mutant disease.

Taken together, these findings are broadly consistent with and confirmatory of signals reported in prior, smaller series. The inclusion of a dedicated BRAF mutation-specific subgroup analysis adds an additional exploratory dimension not systematically addressed in prior surgical LMD literature; however, these subgroup findings should be regarded as hypothesis-generating rather than definitive. Other reported risk factors for LMD, such as tumor resection method (ie, en bloc vs piecemeal), were not associated with LMD development in our cohort. However, prior studies have suggested that en bloc resection is associated with a reduced risk of LMD, potentially due to lower rates of tumor spillage.^7^^,^^16^^,^^36^ These studies included various primary tumor histologies, but melanoma was identified as having a particularly strong association between resection technique and LMD risk. Additionally, unlike the findings of Press et al., our analysis did not show that a cystic or hemorrhagic MBM conferred an increased risk of LMD.^14^ In fact, Press et al found that melanoma histology was not predictive of LMD per se, but hemorrhage was, and hemorrhage was significantly associated with melanoma histology. They concluded that the presence of hemorrhage, rather than histology, is the primary driver of LMD development. The authors proposed that patients with hemorrhagic and/or cystic BMs might benefit from alternative strategies, such as preoperative SRS, to reduce the risk of postoperative LMD.^14^^,^^22^^,^^26^

Although our data suggest that scalp and head and neck primary melanoma sites are associated with a lower risk of LMD compared with upper extremity tumors in BRAF V600-mutated cases, prior studies have reported that scalp melanomas carry a higher risk of MBM development.^40^^,^^41^ A possible explanation for this discrepancy is survival bias. Patients with scalp melanomas often harbor adverse prognostic features such as increased thickness, ulceration, and higher mitotic rate, leading to higher distant failure and overall recurrence rates.^40^ Consequently, fewer patients live long enough to develop LMD. In addition, scalp and head and neck melanomas are frequently detected late, sometimes with brain metastases as a presenting feature, which further shortens survival and may reduce the observed incidence of LMD in this subgroup. Notably, given the potential for false-positive findings arising from multiple comparisons within a relatively small molecularly defined subgroup, these site-specific associations should be regarded as exploratory and warrant confirmation in external datasets.

Moving forward, these findings, together with evidence from prior studies, may help identify patient subgroups at particularly high risk for LMD, including those with BRAF V600-mutant disease, multiple brain metastases, or those undergoing craniotomy (Table 4). Importantly, given the retrospective design and the potential for unmeasured confounding, these findings do not currently support changes in surgical decision-making or routine clinical practice. Rather, they are intended to be hypothesis-generating, identifying patient subgroups who may warrant closer postoperative neurologic and CSF surveillance and who could be prioritized for enrollment in future prospective studies evaluating intraoperative strategies, adjuvant intrathecal chemotherapy, or escalated systemic therapy with BRAF inhibitors in BRAF V600-mutant disease.^19^ Furthermore, while adjuvant WBRT following surgical resection may have mitigated the risk of LMD in the past, it has since fallen out of favor in many settings due to neurocognitive toxicity.^45^^,^^46^ Given the overall poor prognosis of patients with LMD, selective administration of postoperative WBRT in high-risk MBM patients may warrant consideration. Preoperative SRS for brain metastases has also been shown to decrease the risk of nodular LMD.^42-44^

**Table 4.** Risk factors for leptomeningeal disease in patients with brain metastases: present study and prior literature

Risk factor	Association with LMD	Source
BRAF V600 mutation	↑ Risk	Present study; prior studies^6^ ^,^ ^8^ ^,^ ^30–32^
Craniotomy as initial MBM treatment	↑ Risk	Present study; prior studies^7^ ^,^ ^19^ ^,^ ^22-25^
Posterior fossa resection	↑ Risk	Present study (descriptive/univariate only); prior studies^22–25^ ^,^ ^37–39^
Number of brain metastases (BRAF V600-mutant subgroup)	↑ Risk	Present study; prior studies^12-14^ ^,^ ^27^ ^,^ ^33-35^
Proximity to CSF pathways (dural-based/intraventricular metastases)	↑ Risk	Prior studies^19^ ^,^ ^26^
Ventricular opening during resection	↑ Risk	Prior study^19^
Absence of extracranial disease at the time of surgery	↑ Risk	Prior studies^19^ ^,^ ^37^
Increased tumor volume	↑ Risk	Prior study^19^
Younger age	↑ Risk	Prior study^27^
Distant brain failure	↑ Risk	Prior study^27^
Breast and colorectal cancer	↑ Risk	Prior studies^15^ ^,^ ^27^
Piecemeal tumor resection method (vs *en bloc*)	↑ Risk	Prior studies^7^ ^,^ ^16^ ^,^ ^36^
Cystic or hemorrhagic MBM	↑ Risk	Prior studies^14^ ^,^ ^22^ ^,^ ^26^
Primary melanoma site: scalp, head, and neck (vs upper extremity; BRAF V600 subgroup)	↓ Risk	Present study
WBRT post-surgery	↓ Risk	Prior study^46^
Preoperative SRS	↓ Risk	Prior studies^42-44^

Finally, having a BRAF V600 mutation, albeit associated with LMD, holds potential as a therapeutic avenue for LMD. Case reports have documented encouraging responses to BRAF inhibitors such as vemurafenib and dabrafenib in patients with LMD.^47-49^ Notably, one case described a patient with BRAF V600-mutant LMD who achieved a 19-month survival after sequential therapy with vemurafenib, whole-brain radiation, and combined dabrafenib-trametinib treatment.^49^ Larger prospective studies are needed to confirm the safety and efficacy of these agents.

Future investigations should prioritize prospective, multi-institutional cohorts incorporating granular surgical variables, longitudinal systemic therapy exposure, and molecular tumor profiling to clarify the biological and treatment-related determinants of LMD. In this context, emerging CSF liquid biopsy approaches may enable earlier or more sensitive detection of LMD in clinically high-risk patients^50^ and provide insight into the molecular drivers of leptomeningeal progression, although prospective validation is required prior to routine clinical adoption.^51^ Until prevention strategies or validated biomarkers become available, carefully selected clinical scenarios may still offer opportunities for earlier therapeutic intervention and improved outcomes. Finally, standardized subclassification of LMD patterns and integration with contemporary therapeutic strategies will be essential to refine risk stratification and guide future clinical trials.

### Limitations

Despite the strengths of our study, including its large cohort size, which enabled a robust assessment of prognostic factors, several limitations should be acknowledged. First, as a single-institution study, our findings are inherently subject to selection bias, and data accuracy may be affected by potential coding or documentation errors. Moreover, this study’s retrospective design limits our ability to establish causal relationships, and prospective, multi-institutional validation is needed to confirm these findings. Additionally, treatment paradigms for MBM, including targeted therapy, immunotherapy, and radiation strategies, have evolved substantially over the study period, which may further limit external validity across contemporary practice settings. Importantly, residual confounding, particularly confounding by indication for surgical intervention, remains a key limitation. Clinical factors that influence the decision to pursue craniotomy, including tumor location, degree of mass effect, proximity to CSF spaces, and overall intracranial disease burden, are difficult to fully capture in retrospective datasets and may independently contribute to the risk of LMD. Accordingly, the observed association between craniotomy and LMD should be interpreted cautiously and viewed as hypothesis-generating rather than causal.

Because multiple subgroup and site-specific comparisons were performed, particularly within a molecularly defined *BRAF*-mutant subset, the potential for type I error remains, and these findings should be regarded as exploratory pending external validation. Furthermore, we did not subclassify LMD into classic (cLMD) versus nodular (nLMD) patterns, which may have provided additional insights into disease behavior and outcomes. In addition, the diagnosis of LMD in our cohort was primarily based on radiographic criteria, with only a minority of patients undergoing confirmatory CSF analysis, potentially limiting diagnostic certainty. Finally, the introduction of BRAF inhibitors in 2011 substantially altered survival expectations for patients with BRAF-mutant melanoma and may therefore confound observed associations between mutation status and LMD incidence. Future analyses incorporating therapy-era stratification or time-dependent modeling will be important to disentangle biological risk from treatment-mediated effects.

## Conclusion

In this retrospective analysis of 829 patients with melanoma brain metastases, LMD occurred in 16% of cases. BRAF V600 mutations and craniotomy as initial treatment were independently associated with increased LMD risk. Among BRAF-mutant patients, having 4 to 10 brain metastases further elevated this risk. Prospective studies are needed to validate these findings and guide management strategies.

## Data Availability

The data collected for the study, including individual participant data and a data dictionary defining each field in the set, will be made available to researchers upon reasonable request and IRB approval, via sdferguson@mdanderson.org.
